# Adversity, emotion, and resilience among Syrian refugees in the Netherlands

**DOI:** 10.1186/s40359-022-00963-w

**Published:** 2022-11-08

**Authors:** Tengku Nila Fadhlia, Disa A. Sauter, Bertjan Doosje

**Affiliations:** Department of Social Psychology, University of Amsterdam, Amsterdam, The Netherlands

**Keywords:** Adversity, Emotion, Resilience, Syrian, Refugees, Migration

## Abstract

**Background:**

Syrian refugees comprise the vast majority of refugees in the Netherlands. Although some research has been carried out on factors promoting refugee resilience, there have been few empirical studies on the resilience of Syrian refugees.

**Method:**

We used a qualitative method to understand adversity, emotion, and the factors contributing to resilience in Syrian refugees. We interviewed eighteen adult Syrian refugees residing in the Netherlands and used thematic analysis to identify the themes.

**Results:**

We identified themes and organized them into three main parts describing the challenges (pre and post-resettlement), key emotions pertaining to those experiences, and resilience factors. We found six primary protective factors internally and externally promoting participants' resilience: future orientation, coping strategies, social support, opportunities, religiosity, and cultural identity. In addition, positive emotions constituted a key feature of refugees’ resilience.

**Conclusion:**

The results highlight the challenges and emotions in each stage of the Syrian refugees’ journey and the multitude of factors affecting their resilience. Our findings on religiosity and maintaining cultural identity suggest that resilience can be enhanced on a cultural level. So it is worth noting these aspects when designing prevention or intervention programs for Syrian refugees.

**Supplementary Information:**

The online version contains supplementary material available at 10.1186/s40359-022-00963-w.

## Background

“We are Syrians; if it were not for the ruin in our country

we would never consider migration and asylum.” [P13-Male]

As this statement illustrates, war has forced the displacement of many people in Syria. The current civil war in Syria is the worst international humanitarian tragedy since the Second World War [1]. After a decade of crisis, over six million people have fled from Syria, and even more have been internally displaced, making Syrians currently the largest refugee population in the world [2]. Most have sought refuge in neighboring countries, such as Turkey, Jordan, and Lebanon. However, since 2015, around one million Syrian refugees have settled in Western Europe [3].

Given the current circumstances in Syria, the influx of Syrian refugees coming to Europe is not likely to end soon, nor can existing refugees expect to find a safe haven to return to in their home country in the near future. It is, therefore, essential to gain more insights into the experiences of this refugee population. The present study examines the challenges, emotions, and resilience of Syrian refugees living in the Netherlands. Syrian refugees are, by far, the largest group of asylum applicants in the Netherlands, with more than 89.000 requests from 2012 to 2020, of which around ninety percent were approved [4].

Like other refugee groups, Syrian refugees disproportionately suffer from mental health problems. A study of over 3000 Syrian refugees who had been given asylum in the Netherlands found that 41% reported having mental health issues, including anxiety and depression. These levels are much higher than the rate found in the general Dutch population (< 15%) [5]. Refugees are at higher risk of developing mental health problems due to their higher rates of traumatic experiences, including persecution and violence. Moreover, in resettlement, refugees may face additional challenges related to integration into the host country.

The majority of previous studies on refugee mental health showed the high prevalence of mental health problems caused by traumatic experiences relating to war, displacement, and other postmigration variables [see 6, 7]. In the present study, we shift the focus from refugees’ mental health problems to understanding what allows individuals in this population to cope psychologically. This aligns with recent scholarship that has argued that refugee research needs to focus on resilience [8–10] to complement research using a psychopathological approach [11].

Within the literature on marginalized populations exposed to high levels of adversity, there is growing knowledge of the factors that affect resilience. For example, the risk and protective factors of a group of Palestinian women in Gaza can be identified at the individual, family, community, and societal levels, and include family ties, psychosocial resources, and personal resources [12]. Studies on Palestinian refugee children in Gaza and West Bank have pointed to risk factors, including poverty, violence, and marginalization, and key protective factors like youth education, supportive relationships, and social participation [13]. Moreover, feelings of insecurity from the social environment have been found to affect Palestinian children’s mental health and psychological functioning [14]. Research on refugees and asylum seekers in transitional countries has identified social support, cognitive strategies, education and training opportunities, employment and economic activities, behavioral strategies, political advocacy, environmental conditions, religion, and spirituality as the factors promoting their well-being [15]. Thus, multiple protective and risk factors of refugees’ resilience in different ecological domains have been identified across migration stages. Identifying and organizing these factors using a theoretical framework will benefit attempts to design intervention strategies and policies for refugees.

The present study sought to understand resilience in Syrian refugees after their resettlement in a western-European country. This entails a different set of challenges than refugees displaced internally or in neighboring countries (e.g., Jordan). Coming from a different cultural and geographical background means that Syrian refugees resettling in a western-European country face challenges including culture shock, adjusting to a different climate, discrimination, and language barriers [16, 17]. Language difficulties in particular lead to problems with making social contact with the majority culture and finding employment, which in turn can affect resilience [17].

Resilience is generally seen as a process [18] by which individuals navigate their way to acquire multiple resources that sustain or even improve their wellbeing in contexts of adversity [19]. Resilience research has evolved from identifying internal resource resiliency factors (trait resilience) to also including external socioecological factors [20], such as family, friends, and community [12, 21]. Socioecological models of resilience have emphasized the intertwined relationship between individual and the social and ecological systems in which they exist [20]. However the socioecological framework of resilience has not been widely used with refugee populations. Further investigations are thus needed to examine refugees’ resilience while considering a wide range of potentially protective factors at different levels. This is especially important because the concept of resilience for Syrian refugees includes communal coping strategies, such as familial and social relationships, Syrian community assistance, and religion [22].

## The current study

Although some research has been carried out on factors promoting refugee resilience, there have been few empirical investigations into Syrian refugees’ resilience. The few studies that have been conducted to date point to the importance of employment status, educational level, living inside or outside a refugee camp, income, social support, and faith [16, 22–25]. The current study seeks to add to this scarce but growing body of literature on Syrian refugees’ resilience. Specifically, the primary purpose of the present study was to develop an understanding of factors contributing to the resilience of Syrian refugees in the Netherlands using a qualitative method. The study about the determinants of Syrian refugees’ resilience listed above was mainly based on quantitative data. Investigating Syrian refugees’ resilience using a qualitative approach can allow for discoveries of unknown protective factors that may emerge from the lived experience of the participants [26]. Furthermore, the qualitative method can give power to minority and marginalized voices, which is important in the study of resilience [26]. In addition, we examine adversity and emotion patterns throughout the four stages of the migration journey: the flight from Syria, transition period, asylum center in the Netherlands, and resettlement in one’s own house.

Only one previous study [22] considered the pre and post-resettlement trajectory in understanding Syrian refugees’ resilience; no study to date has explored emotions in this context. We thereby take into account the adversity refugees were exposed to during each of the stages of migration and the emotions pertaining to each stage. The adverse situation is needed to provide context to resilience [11, 27], and emotions might give us insight into their experiences and resilience. Finally, in this study, we identify the factors of refugees’ resilience at multiple levels, following Ungar & Theron [19], to consider the ecological contexts as well as individual-level factors that shape resilience.

## Method

### Design

This was an exploratory study using a qualitative design (Additional file 3). Using a qualitative approach in exploring Syrian refugees’ resilience from their own narrative can allow for discoveries of unknown protective factors that may emerge from the lived experience of the participants [26]. Furthermore, the qualitative method may empower minority and marginalized voices, which is critical in the field of resilience research [26].

The primary purpose of this study was to develop an understanding of factors contributing to the resilience of Syrian refugees in the Netherlands. We considered the adversity they endured during each migration phase to give context to their resilience. We were also interested in looking at the emotions related to each stage to understand their experiences and resilience better.

### Participants

Eighteen interviews were conducted with Syrian refugees residing in the Netherlands. The participants were ten men and eight women with a mean age of 29 years (age range 18–41, *SD* = 6.91). They had been in the Netherlands for an average of 3.2 years (*SD* = 2.2), with the average time spent in an asylum center being 7.2 months (*SD* = 5.51). Demographic information is shown in Table 1.

**Table 1:** Participants’ demographic information (N = 18)

Demographic Information	Value
Age at interview (years; mean, range)	29 (18–41)
Length of stay in the Netherlands (years; mean)	3.2
Length of stay in asylum center (months; mean)	7.2
*Gender*	
Male	8
Female	10
*Marital Status*	
Single	8
Married	7
In a relationship	2
Separated	1
*Highest education*	
High school	5
Bachelor	9
Master	4
*Religion*	
Islam	15
No affiliation	3
*Residence status*	
Temporary	15
Permanent	3
*Employment*	
Full time	1
Part-time	1
No employment	7
Student	9

### Procedure

The participants were recruited using the snowball sampling technique based on personal networks and social media. Two participants were personal contacts of a colleague, and the others responded to a research advertisement posted in a social media group for Syrian people in the Netherlands. The interviews took place between June and August 2020. All of the interviews were conducted in English by the first author, a Muslim, and thus has a similar religious background to the majority of the participants. Moreover, the interviewer has experience working with refugees, which facilitated establishing a trusting relationship with the participants. We used Skype for Business due to precautions relating to in-person meetings due to the COVID-19 pandemic. Some minor challenges from the online interview format were signal quality and participants’ lack of familiarity with the platform used.

The interview was semi-structured to examine the perception of protective factors that affect Syrian refugees’ resilience. The participants were asked about their life in Syria, their migration story, their experience after coming to the Netherlands, their coping strategies, the factors contributing to developing their resilience, their perception of resilient people, and their hopes for the future (Additional file 1). The duration of the interviews ranged from 45 to 55 min. All of the participants gave informed electronic consent before the interviews. Afterward, each participant received 10 Euros for sharing their valuable time and information, though several participants refused to accept the money. The interviews were directly recorded using the recording function on Skype for Business and then transcribed by the first author.

### Analysis

All interviews were transcribed verbatim, and any personal identification of the participants was removed to ensure privacy. The 293 pages of interview transcripts were transferred into MAXQDA version 20 [28], with which the coding was performed. Data were analyzed using the thematic analysis method as formulated by Braun and Clark [29]. The first step of the analysis was to read through the transcripts as a data familiarization process. We used both deductive and inductive approaches in the coding process. Initially, a coding scheme (Additional file 2) was built based on a review of previous literature, which was then piloted on several transcripts. To establish an inter-coder agreement, we randomly selected three interviews to be double-coded. As there were 18 interviews, this made up 16.7% of the data set. The first author and a trained research assistant acted as the two independent coders. They both coded the selected three transcripts, discussed the coding, and then adjusted the coding scheme. In this process, we added new codes or renamed existing codes to facilitate the coding of recurring topics which were relevant to the research questions. Any disagreements were discussed until mutual agreement was reached. After discussion, the inter-coder agreement was calculated using MAXQDA, yielding a 0.89 Kappa score, as displayed in Table 2.

**Table 2:** Kappa score calculation

	Rater 1		Row marginals	
	1^a^	0^b^		
Rater 2				
1^a^	a = 444	b = 28	472	
0^b^	c = 26	0	26	
Column marginals	470	28	498	
P (observed)				0.89
P (chance)				0.01
Kappa				0.89^c^

^a^1 = perfect agreement between two raters

^b^0 = parity with chance

^c^Kappa score calculated from (P_observed_–P_chance_)/(1–P_chance_)

After the coding process, we developed the themes from the data in three phases. First, we searched for themes by identifying similarities and connections between the codes. Next, we reviewed the developing themes to check whether the themes could capture information pertinent to our research question. Finally, we gave names and descriptions of the themes to distill what was distinctive about each theme. A theme provides a unifying framework to describe the data and represents patterns in telling a coherent story about the data [30].

## Results

The data is organized into ten themes, as shown in Table 3. Two themes pertain to the adversities participants had experienced, the next two describe their emotions, and six themes concern factors promoting Syrian refugees’ resilience.

**Table 3:** Themes generated from the interviews

Themes	Sub-themes
*Adversity*	
Theme 1: Pre-resettlement adversity	War-related adversity Migration-related adversity
Theme 2: Post-resettlement challenges	Asylum-related adversity Challenges in navigating a new life
*Emotions*	
Theme 3: Key emotions pre-resettlement	Fear, sadness, and anger
Theme 4: Key emotions post-resettlement	Happiness, hope, and gratitude
*Resilience*	
Theme 5: Future orientation	Hope for the future
	Meaning in life
	Maintaining healthy lifestyles
Theme 6: Coping strategies	Problem-focused
	Emotion-focused
	Dysfunctional coping
Theme 7: Social support	Family and friends support
	Community support
	Social connection
Theme 8: Opportunities	Sense of safety
	Opportunity
	Contribution
	Mastering the Dutch language
	Positive life events
Theme 9: Religiosity	Experiential
	Ritualistic
	Ideological
	Intellectual
Theme 10: Cultural identity	Maintaining cultural identity

### Adversity

#### Theme 1: Pre-resettlement adversity

This theme maps the participants’ traumatic war experiences and adversities during migration. In Syria, the refugee participants experienced torture, assault, destruction of their homes and villages, witnessing physical violence against friends and family members, the death of family members and friends, and a lack of basic supplies. During the war, the refugee participants experienced bombs and military airstrikes that devastated their homes and villages and damaged key infrastructure. ISIS [The Islamic State of Iraq and Syria] besieged their cities, and no one could go in or out. Many participants lost family members and friends. Two participants witnessed physical violence or assault by the military force on family members and friends or acquaintances. Another traumatic experience during the war was living without electricity, food, and other essentials. In one participant’s words:

The city lived under siege and lacked food for more than three and a half years. I know some people who were my age and died because of starving, the media wasn't covering that time a lot . . . And that made me feel like going through a terrible time. And no electricity, no connections that were even worse. But what I found it I counted until now, and the worst experience is when I saw my little sister . . . said that she's hungry . . . and I felt that I'm useless. [P12-Male]

Participants had to flee to a neighboring country to seek refuge; all of our participants went to Turkey. There, they faced other traumatic experiences, such as worker exploitation, physical violence, and threats to their lives. The most difficult challenge was racism, and chronic discrimination, which they said happened to almost all Syrian people. Chronic discrimination limited their economic and social opportunities. They could not get a work permit, so they had to work illegally. This meant that they worked maximum hours for minimal pay, resulting in financial strain. Also, getting a job without a residence permit was difficult, so they lived illegally and tried to do everything they could to survive.

In Turkey, you don't have a residence card. You are living illegally. So you don't have any right to work or go to school or learn the language or anything. So the day you work you can eat, the other day if you don't work, you cannot eat. We tried our best to stay in Turkey because it was near Syria and it was an Islamic country, but it was really difficult, and we faced many problems. Not only financial problems but also problems with people there. They don’t accept strangers or foreigners. [P5-Female]

#### Theme 2: Post-resettlement challenges

This theme outlines the participants’ experiences after they arrived in the Netherlands. First, they had to spend some time in an asylum center before being assigned to their own house. Most participants thought they would only spend a short time living in the asylum center, but there was a longer waiting period than expected. Some participants said that boredom always accompanied them since they did not find the limited activities helpful. Safety was another problem that the participants encountered in the asylum center. One participant witnessed women and children being abused by their family members there. For example, husbands abused their wives, and adults abused children. Half of the participants experienced bureaucratic barriers during the asylum process, which affected their waiting period in the asylum center. One participant said, “I did the procedures and waited. I didn't get the status at once… And 25 days later, I got it. But it wasn't as expected even though it was quite clear that I had all the documents” [P1-Female].

After the asylum process was finished and the participants got their residence permits allowing them to live in the Netherlands, they embarked on a new stage of life. They were resettled into their accommodation and started integrating into the new society. However, the participants faced some challenges while navigating their new life in the Netherlands. More than 60% of participants reported having problems adjusting to the new culture, typically related to adapting to new houses and surroundings, the climate, food, making friends, trying to fit into the Dutch community, and culture shock.

Another challenge was language difficulty. Half of the participants said Dutch was not an easy language to learn, and they were still struggling to master it. Participants also suffered from various psychological problems due to war and forced migration. Almost half of the participants mentioned how complex they found the system in the Netherlands when they wanted to work or continue their education. Another difficulty was that their pre-existing qualifications were not recognized in the Netherlands. This prevented them from getting a job matching their qualifications. As one participant, who was already a licensed pharmacist in Syria, explained:

And then, as I noticed it, there was no opportunity, no job as a pharmacist . . . Yeah as a pharmacist even after I qualified with my diploma, to qualify a diploma costs about four years. I have to study something called AKV. It's a sort of examination for pharmacists and doctors, you know; after I passed our AKV, I have to study pre-master for one year, then three years master. So it costs about four to five years, then I can work as a pharmacist. [P7-Male]

### Emotions

#### Theme 3: Key emotions in the pre-resettlement period

This theme outlines the key emotions of our participants in the pre-resettlement phase. When living in Syria before fleeing, fear was an everyday emotion for most participants. In our study, many participants experienced the unexpected loss of family members and valued belongings and a loss of their sense of safety. They were continuously worried about whether they could survive the next day and if they could escape the danger. One participant described a constant fear of losing someone or something he loved, as well as the challenge of displacement.

And that was very tough for me. Also, the fact that I was always feeling afraid because of the things that are going over there and the situation and the bombing and feeling afraid to lose one of my family or my friends, I had to lose my place . . . we have been displaced more than like 13 times. And as you know, like for me personally, I hate moving from one place to another. I just find it hard. So I have done it a lot of times, and that’s very bad. [P12-Male]

When the participants left Syria, they felt sad because they had to leave behind their home, family, and memories. One participant said he felt lonely then, leaving his home and imagining living in a completely new place. In contrast, the dominant emotions in the transit countries were fear and anger. Their illegal status and lack of opportunities scared the participants, forcing them to build temporary survival strategies, for example, taking any job even if it was underpaid. Anger was caused by racism and chronic discrimination. They also felt that they were taken advantage of and used for cheap labor due to their illegal status. One participant said, “it was not easy. Because we were from one to two places, we had to deal with the people, maybe rude and bad people. We suffered a lot. You know, until we came to the Netherlands” [P17-Female].

#### Theme 4: Key emotions in the post-resettlement period

Living in an asylum center in the Netherlands was difficult for our participants. Hence, the dominant emotions in this phase were anger and fear. Participants were angry when they had to deal with the bureaucratic barriers and wait longer than expected in the asylum center. The long waiting periods increased participants’ fear of the uncertainty of their future. Participants were wondering when they could start a normal life and what would happen with their future.

You still have the feeling of that you're going to the unknown, or you are feeling unstable, or you're feeling like stressed about what will happen next, and yeah, it's just mixed of the feeling of you are, I feel unsettled because you never know what will happen next. [P10-Female]

Once the asylum application of our participants was granted, they began a new phase as they moved into their own houses. The dominant emotion in this phase were three positive emotions: happiness, hope, and gratitude. One participant stated that she was happy because she felt accepted in the Netherlands, even though she came from a different culture. Being accepted by society created hope for the participants: “here, people are much more accepting. And that that also kind of makes a person happy and hopeful for the future” [P6-Male].

Another positive emotion mentioned concerning the period after the participants finished their asylum process was gratitude. They felt grateful to live in a safe country and for all the challenges they had been through, which made them stronger. Gratitude also contributed to the hope of our refugee participants, which could serve as a source of their resilience: “so, I am grateful. I'm grateful. So my best is something for the Netherlands. This is what gives me hope for the ideal of the future” [P5-Female].

### Resilience

We argue that the last themes relate to resilience and all periods, especially the post-resettlement period. Given the centrality of emotions to resilience, we believe it is worthwhile to include them in our analysis as an additional category. These pathways between emotions and resilience are of course are not mutually exclusive. Evidence from previous research has showed that positive emotions are the result of being resilient [31], but that they can also aid individuals to use their resources to cope with difficult life events [32].

#### Theme 5: Future orientation

The most frequent topic mentioned under this theme was hope for the future. For the participants, it was essential to have a plan for the future, so they had something to work towards, which gave them hope. All participants shared similar plans for their future, either working or studying. Additionally, the family was a source of hope for most of them.

A second thing is how my parents are at the moment gives me hope for the future. I see their content. I see them happy. I see them not overly anxious anymore. So, that also strengthens one's resolve . . . gives me hope for the future. And of course, another thing is how I see all the people around me . . . I see how everyone is moving on with their lives, despite what they have been through. I see how everyone is growing up and how everyone is flourishing in this new environment. [P6-Male]

Besides hope, the participants also stated that having meaning in life is a source of their resilience. Participants viewed meaning in life as goal-directedness or purposefulness. It helps create an orientation towards the future by giving a reason for actions and consequences. What gave them a sense of meaning in their life is similar to what gave them hope: family. Therefore, we categorized future orientation as a family-level factor. In addition, maintaining a healthy lifestyle was also an aspect of the future orientation. Many participants started to develop healthy habits in the Netherlands, giving them positive feelings about themselves.

#### Theme 6: Coping strategies

Our participants implemented three coping strategies: problem-focused, emotion-focused, and dysfunctional coping [33]. Coping is considered an individual-level factor. In terms of problem-focused coping, half of the participants chose to take action and make plans to try to cope with their challenges directly. The emotion-focused strategy that the participants used most was cognitive reappraisal, that is, trying to reframe the situation in their mind: “... because I had experienced in the Syria war experience let me say, yeah. By remembering the difficulty in Syria, I will consider every other difficulty is nothing... “ [P7-Male]. Participants who engaged in dysfunctional coping used self-distraction. They always tried to keep themselves busy to distract themselves from thinking about their problems.

#### Theme 7: Social support

Social support contributed to participants’ resilience, with support primarily from family and friends but also the broader community. Hence, this is a family and community level factor. Having social connections (the experience of feeling close and connected to others) helped participants buffer the negative impact of their trauma experiences. Social support from family and friends was crucial for almost all participants in terms of both instrumental (i.e., practical) and emotional support.

I think the family supports it. Like yeah, nowadays I feel very, very comfortable after talking to my sister's sharing our ideas like talking about what makes you, what points in life makes you feel anxious and what you want to improve in yourself, and what difficulties you have. Having support from your family and having some friends really helps . . . So surround yourself with positive people, and people are that are willing to share positive ideas. [P14-Female]

#### Theme 8: Opportunities

On the broader society level, having opportunities to develop and grow in a new country was essential to our refugee participants. The fact that the host country provided such opportunities aided their resilience. The chance to build a new future and be able to dream again made the participants optimistic. A sense of safety was the first thing that participants noted as a foundation for other opportunities. War experiences had made them greatly appreciate security. Furthermore, the participants had previous knowledge and skills that they wanted to use to contribute to their new society.

The ability to be actively functioning and participating in society helps me feel like I am doing something that may be important . . . somehow you feel like I am giving back . . . The ability that I am able to serve someone that I am to help someone to support someone in many, like big and small shapes and forms, yet that gives me a sense of achievement or like an accomplishment in my life. [P10-Female]

Mastering the Dutch language was crucial as it dramatically increased opportunities for participants. One participant said that he had worked hard to master the language, and eventually, it paid off. Experiencing positive things in their early time in the Netherlands was also one form of opportunity. One participant had a good experience dealing with COA [Central Agency for the Reception of Asylum Seekers], and he also got the chance to volunteer with them, which gave him a positive perception of the Dutch people.

#### Theme 9: Religiosity

Having faith was one source of participants’ resilience. Believing in God comforted them and helped them make sense of their ordeals. Some of our participants felt that God has a reason behind everything that happens in their life and that God must have a good plan for everyone. Primarily, our participants hold Islamic beliefs, and doing rituals in private space, such as praying five times a day and reciting the Qur'an, relieved them from stress. We place religiosity at the individual level since our participants generally did not mention religion in relation to community, as seen in this excerpt:

I thank God, a believer, and that is very important to me because I can always return to and rely on it. Even though being alone for all these years since I am here alone in this country, if you have God by your side, you somehow feel you're not alone or something . . . based on my religion and belief, there is always a reason behind anything. And there is nothing just like, out of coincidence or something . . . God has a better plan for me. So that feeling gives me a sense of like to be grounded to be settled to be safe. [P10-Female]

#### Theme 10: Maintaining cultural identity

The participants tried to retain part of their Syrian identity in their acculturation process. They wanted to be acknowledged as Syrians and maintain their norms and values while at the same time learning a new culture. We consider cultural identity a community-level factor as it typically involves social activities in larger groups. Practicing religious rituals and beliefs, speaking the Arabic language, making Syrian food, and following cultural traditions are ways the refugees sought to preserve their cultural identity.

Yeah, it was like, because it was also like coming from the idea that I have certain principles (religion, traditions). I don't want to let it go. But at the same time, I want to integrate into the society, and I think upon and to have a place for me . . . I came to that conclusion like okay, this is what I want and like; what is stuff that I cannot compromise, and what? So, and then that moment, it was like really clear to me that what they really want and what are the principles . . . [P3-Female]

## Discussion

This study examined factors contributing to the resilience of Syrian refugees resettled in the Netherlands. To give context to participants’ resilience, we provided a complete story of their adversity. Hence, we organized the themes into three main parts describing the challenges (pre and post-resettlement), key emotions pertaining to those experiences, and resilience factors. We first summarize the adversity and emotional patterns throughout the migration journey and then discuss the factors promoting the Syrian refugees’ resilience.

### Adversity and emotions throughout the migration journey

In Syria, war-related traumatic experiences inflicted fear on participants’ everyday life, as they felt constant fear of losing their loved ones. In our study, many participants experienced the loss of family members and belongings and a loss of their sense of safety. Next, leaving homeland, friends, and family resulted in feelings of sadness. This sorrow turned into fear and anger during the time spent in transit countries. Their illegal status and lack of opportunities made the participants feel uncared for and forced them to build temporary survival strategies. Their anger was caused by racism and chronic discrimination.

Living in an asylum center in the Netherlands was also difficult for the participants. They experienced challenging circumstances, including poor physical conditions at the asylum centers and a lack of safety, as well as bureaucratic barriers and long waits. These conditions elicited anger, and the long waiting periods also increased participants’ fear of the uncertainty of their future. In the final phase, our participants reported that adapting to a host country was stressful, requiring physical, psychological, and socio-cultural adaptation. The refugee participants struggled to adjust to the Dutch culture and Dutch weather. However, positive emotions prevailed after resettling to their own house. Despite the challenges in navigating their new lives, our participants felt grateful to live in a safe country, and they perceived the challenges that they had been through only made them stronger. Being accepted in the Netherlands made our participants happy and created hope for the future. The participants also experienced gratitude, which may have contributed to their resilience.

### Factors promoting resilience

**Fig. 1:**
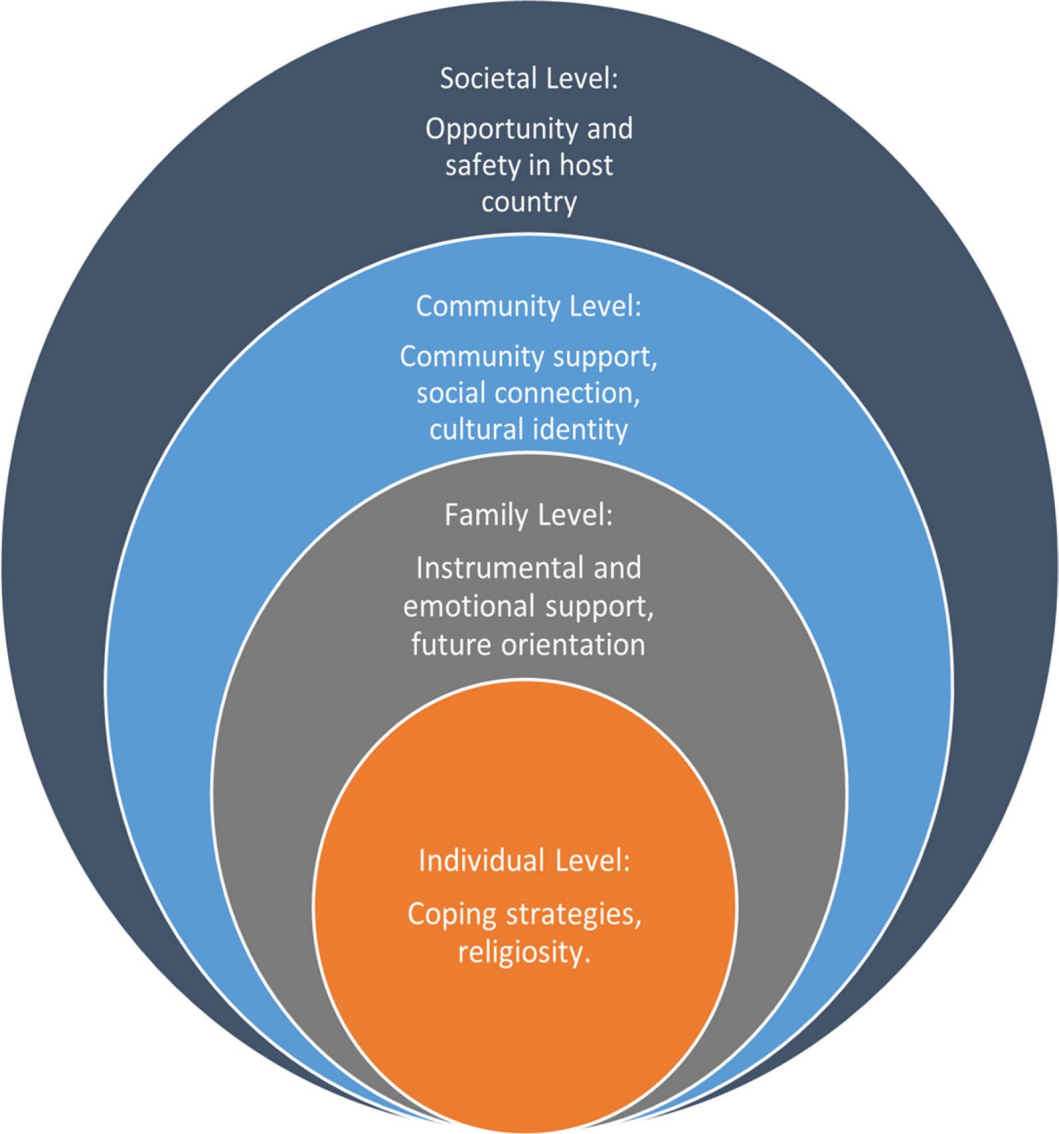
Factors promoting refugees' resilience. This figure shows that resilience is affected by factors at multiple levels: individual, family, community, and larger society

Our participants had undergone highly stressful experiences in every phase of their forced migration. These experiences are atypical in human life since the levels of stress they have experienced go beyond common frustrations and may not be endurable for every human being. Nevertheless, our refugee participants managed to cope. They showed an ability to utilize internal and external factors to protect them from the most detrimental effects of forced migration. Our results support the idea of Ungar and Theron [19] that resilience is a process in which individuals and social factors (at the family, community, and societal level) contribute to the development of resilience. Figure 1 shows the factors promoting Syrian refugees’ resilience in this study.

### Individual level factors

At the individual level, our findings point to coping strategies involving skills and personal values as key to resilience. Coping relates to the approaches and personal abilities that people employ when facing difficult circumstances in life and has been highlighted as a resiliency factor among refugees in previous work. For example, a study of the internal resiliency factors of refugees in Canada found that positive coping strategies could be strengthened by helping refugees reframe past difficulties and highlighting their strengths [34].

Our refugee participants utilized problem-focused, emotion-focused, and dysfunctional coping strategies. Refugees employed different coping strategies at various stages of their migration journey. Emotion-focused coping is more likely to occur when it is perceived that nothing can be done to modify the challenging environmental conditions [35]. Our participants indeed mostly used this coping strategy during the transit and asylum period when they essentially felt hopeless. On the other hand, problem-focused forms of coping are more probable when conditions are appraised as amenable to change ([36]. Our participants used this strategy during the resettlement period after they had finished the asylum process.

We consider religiosity an individual-level resiliency factor since our data did not point to a social function of religiosity. For our participants, believing in God and practicing individual religious rituals such as praying helped them get through difficult times. This finding aligns with previous research, which has shown that religious coping contributes to resilience among adult Muslim refugees for whom religion can be a source of solace and comfort in times of adversity [37]. This is different from many Western societies; religion and its rituals are important in Middle Eastern cultures [3], and this is how people make meaning from tragic life events [38]. Moreover, in Islamic teaching, there is a universal belief that all things happen to the will of Allah [39], which affects how our participants, predominantly Islamic believers, interpret their suffering and cope with their difficult circumstances.

Faith is also seen as a two-way relationship with God in which one would be rewarded for following God’s rules [16]. Thus, suffering is considered a path to personal and spiritual transcendence, which differs from the Western trauma approach that aims to alleviate pain and restore pleasure [38].

### Family level factors

We found that having family support was vital for resilience. This finding is in line with the findings of previous studies [21] that showed family support as a source of resilience outside the individual level. Our results divide family support into two categories, instrumental and emotional support. Instrumental support involved receiving material aid from family members to meet basic needs. However, the family was especially crucial for emotional support: encouragement, motivation, wise words, and comfort to help during difficult times.

Orientation for the future is another factor that helped our participants reduce feelings of stress and hopelessness. We considered orientation for the future also at the family level because the family was a primary source of their hopes and the one that gave them meaning in life. The presence of family was essential in participants’ lives, as family motivated them to continue with their lives and rebuild their dreams, despite everything they had been through. This is in line with the previous finding that having a strong family to nuclear and extended family is important for refugees as it can provide a sense of agency. Having strong family ties results in family support when needed, and it makes refugees experience that they could handle the difficulties at that given time [12]. In collectivist cultures, the interconnectedness of self and others and the need to maintain positive relationships with family are strongly valued [40]. Therefore, family and close friends were crucial for our participants.

### Community level factors

The community can contribute as a source of resilience by providing individuals with the required resources. Community support gave our participants a sense that they were not alone and created social connections, a feeling that they belonged to a group. Community support is essential in promoting refugees’ resilience since it provides company, safety, reassurance, and validation of their feelings [27, 41]. Our participants received support from the Syrian community in the Netherlands in terms of both emotional and instrumental support. Digital support was also available for our participants. For example, our participants pointed out that a Facebook group of Syrian communities in the Netherlands provides information about education, health, housing, etc. In line with previous research [25], digital social support from the Syrian community serves as a resilience source for Syrian refugees in the Netherlands.

Another community-level factor that our participants viewed as one of the sources of their resilience was cultural identity, that is, maintaining their Syrian identity in the Netherlands while integrating with the new host culture. Our participants kept their cultural identity by practicing religious rituals and beliefs, speaking Arabic, cooking Syrian food, and participating in cultural traditions. This result is consistent with a study [42], which found that higher Syrian identification was associated with lower symptoms of depression and anxiety. A Syrian identity could give a sense of control, uniqueness, and meaningfulness amidst the challenges of navigating a new life in the host country.

### Societal level factors

Lastly, we found resiliency factors that occurred at the societal level: safety and opportunities in the host country. Our participants greatly appreciate now living in a safe country, which was something they had missed in Syria. A sense of safety was a foundation for other opportunities. This is consistent with studies that have found that feeling safe is a key protective factor for refugees’ resilience [14, 22]. Our participants felt that the Netherlands provided a great deal of opportunities, especially for those who have mastered the language. Refugees’ capacity and opportunities offered by society must be combined to allow integration into the host society [43]. Our refugee participants possessed skills and knowledge, and when they had the chance to contribute, they felt they had something valuable to offer. However, the Dutch labor market requires formal qualifications to obtain a job, and a foreign qualification is often valued at a lower level in the Netherlands [43]. As a result, our participants were forced to take jobs below their qualifications or repeat their education.

### The relationship between positive emotions and resilience

We identified three positive emotions as highly relevant in refugees’ resettlement phase: happiness, hope, and gratitude. Studies have shown that individuals with higher levels of psychological resilience display more positive emotions [44]. Experiences of positive emotions are one of the active components that protect individuals from psychological problems in the aftermath of catastrophes [31]. For our participants, happiness came from feeling accepted by the new society even though they came from a different culture. It is not easy for newcomers to adjust to an alien society, especially having had to flee their homeland.

Being in a safe country resulted in two primary positive emotions: gratitude and hope. Our participants were grateful to live in a country where they could sleep at night without worrying about a bomb attack and felt full of hope about the future. Furthermore, acceptance from the host society created hope for our participants. They felt hopeful that they could build their future in the Netherlands. This is in line with previous research that found that gratitude was one of the most frequent positive emotions experienced by people following the September 11th attack in America and that resilience was positively correlated with hope [31].

Notably, positive emotions are directly positively associated with resilience, as well as being indirectly associated with resilience via coping strategies, particularly adaptive coping [45]. Resilient individuals are argued to experience positive emotions in the midst of adversity, which enables them to thrive and benefit from positive outcomes [46]. Over time, the experience of positive emotions functions to assist high-resilient individuals in their ability to recover effectively from daily stress [32]. Individuals who experience frequent positive affect thrive through various difficult events not simply because they feel good, but because they have resources at their disposal to deal with the difficulties [47].

### Limitations

This study is not without limitations. In particular, there is a bias in participant selection. Our sample was limited to participants fluent in English, which might have excluded Syrian refugees with a lower socioeconomic status. Moreover, neither the interviewer nor the participants were native English speakers, and consequently, the verbal expressions were sometimes limited during the interview. Another limitation is that this study involved sharing details about the participants' difficult life experiences. This means that our sample might consist of quite resilient individuals who were willing and able to take part in such a study, thus it is difficult to comment on individual difference in resilience based on our findings. Finally, as is the case for most qualitative research, our small sample size and lack of statistical analyses of quantitative data limit the generalization of the findings to the broader (refugee) population. It is therefore future research in quantitative large-scale studies are needed to address these limitations.

### Practical implications

The present findings have some important practical implications. Our participants noted that the Dutch government paid little attention to their mental health problems, especially when staying in the asylum centers. Our findings also show that waiting in asylum centers was challenging for participants due to preventable factors like crowding, lack of suitable activities and support, and administrative delays and complexity. It is crucial to design programs to increase resiliency in refugees, including providing a safe and positive environment for refugees in the asylum centers. Options might include specific room arrangements for families or involving refugees in designing activities according to their needs and preferences. Our findings on religiosity and maintaining cultural identity suggest that resilience can be enhanced on a cultural level. So it is worth noting these aspects when designing prevention or intervention programs for Syrian refugees.

## Conclusions

In conclusion, this qualitative study describes resilience factors of Syrian refugees resettling in the Netherlands. We organized the findings into ten themes portraying participants' resilience journey, emotions, and a range of resilience factors. The findings suggest that individual, family, community, and societal factors promote resilience for Syrian refugees. The results highlight the challenges and emotions in each stage of the Syrian refugees’ journey and the multitude of factors affecting their resilience. A large-scale follow-up study with a quantitative method could provide insights into how these factors interact, including the role of positive emotions. We hope that the findings of this study will contribute to the formulation of a framework for psychological assessment and intervention, and civic integration policy based on the need of Syrian refugees, thereby enhancing the quality of psychological and social wellbeing of this population.

## Supplementary Information

**Additional file 1**. The Interview Protocol.

**Additional file 2**. The Coding Scheme.

**Additional file 3**. The 32 Items COREQ Checklist.

## Data Availability

The data are not publicly available to protect participants’ privacy but are available from the corresponding author on reasonable request.

## Abbreviations

ISIS: The Islamic State of Iraq and Syria
AKV: *Algemene Kennis en Vaardighedentoets*
COA: Central Agency for the Reception of Asylum Seekers
