# Supplementary material for: Adversity, emotion, and resilience among Syrian refugees in the Netherlands
Source: BMC Psychol. 2022 Nov 8;10:257. doi: 10.1186/s40359-022-00963-w (PMC9643972; doi:10.1186/s40359-022-00963-w)
Supplement: Supplementary file 1 — Additional file 1. The Interview Protocol. [file 40359_2022_963_MOESM1_ESM.docx]

**Interview Protocol**

**Complete checklist prior to interview:**

□ Obtain written consent

□ Gather demographic information

□ Turn on recording function

**Interviewer:**

I am going to ask you some questions about your life after coming to the Netherlands. As you may find differences about your life in here with previous one in your home country, you may or may not experience some difficulties to adjust. We are trying to learn about what helps people during times of difficulty in their life. If any of the questions are not clear, please tell me and I will try to ask it in a different way. Once I ask you the question, you can answer for as long as you would like, sharing your experiences as a Syrian person living in the Netherlands. Do you have any questions before we start?

**Interviewer:**

Before prompting the first questions, please introduce yourself and tell a bit about yourself. [Try to talk about something in common with participant as a building rapport (especially about experience as a new comer in the Netherlands)]

**Interviewer:**

Could you tell me about the reason for your migration? What was the migration process like for you? Again, if you’d rather prefer not to discuss this, feel free to not respond and we continue.

**Interviewer:**

Could you tell me about your experiences after coming to the Netherlands? How was that time for you and your family?

**Interviewer:**

What do you do when you face difficulties in your life? (probe about the coping strategy)

How do you feel when facing difficulties?

How do you make meaning of difficulties in your life?

What do you learn from difficulties in your life?

**Interviewer:**

What contributed to your sense of wellness and mental health in this situation? In other words, what makes you feel happy and healthy now?

**Interviewer:**

How do you describe people who live well in a situation despite the many problems they face? Do they have certain characteristics?

**Interviewer:**

Describe what gives you hope for the future? How does an ideal future for you look like?

Interviewer: This is the end of the interview. Is there anything that you would like to add?

Before I end the interview and explain a little bit more about what we are going to do, I’d like to ask you whether you have any comments or questions about the interview and the study?

___________________________________________________________________________

**Debriefing**

**Purpose**

Thank you very much for participating in the interview. The purpose of this interview was to learn about how Syrian people live well in time of difficulties. Your participation is highly valued.

**Confidentiality**

As said before, this interview will be transcribed and any identifying information will be removed from the transcription. Once transcribed, the recording will be deleted. Your personal information will be kept strictly confidential and stored in a secure location to ensure your privacy.

**Final Report**

If you are interested in the results of this study, you can request a summary of the study by emailing the lead researcher.

Thank you.
