# Supplementary material for: Adversity, emotion, and resilience among Syrian refugees in the Netherlands
Source: BMC Psychol. 2022 Nov 8;10:257. doi: 10.1186/s40359-022-00963-w (PMC9643972; doi:10.1186/s40359-022-00963-w)
Supplement: Supplementary file 2 — Additional file 2. The Coding Scheme. [file 40359_2022_963_MOESM2_ESM.docx]

**Coding Scheme**

| Migration Stages | Specification | Coding Option |
| --- | --- | --- |
| Life in Syria | Traumatic experience | 1. Lack of food/electricity/other supplies |
|  |  | 1. Destruction of homes and livelihoods/war situation |
|  |  | 1. Torture/physical violence/ assault |
|  |  | 1. Sexual violence |
|  |  | 1. Witnessing physical violence or assault |
|  |  | 1. Loss or disappearance of loved ones/friends/relatives |
|  |  | 1. Other frightening situation where someone’s life in danger |
|  | SES background | 1. Student |
|  |  | 1. Paid/voluntary work |
|  |  | 1. Unemployed |
|  | Perceived emotion about life in Syria | 1. Positive emotion |
|  |  | 1. Negative emotion |
|  |  | 1. Mixed |
| Reason for migration | Push factors | 1. Political and security situation in home country |
|  |  | 1. Persecution (religious/political) |
|  |  | 1. The outlook for future |
|  | Pull factors | 1. Asylum seeker policies in destination countries |
|  |  | 1. Perceptions of destination countries’ acceptance of refugees |
|  | Reason to choose NL | 1. Actively |
|  |  | 1. Randomly/Forced |
|  | Perceived emotion when leaving Syria | 1. Positive emotion |
|  |  | 1. Negative emotion |
|  |  | 1. Mixed |
| The journey/life in transit country | Traumatic experience | 1. Physical violence |
|  |  | 1. Threatened life |
|  |  | 1. Work exploitation |
|  |  | 1. Forced separation from family or close friends |
|  | Discrimination experience | Chronic discrimination (any kind of discrimination, like treated less respect, less courtesy, insulted, harassed) |
|  | Perceived emotion in transit life | 1. Positive emotion 2. Negative emotion |
|  |  | 1. Mixed |
|  | Perceived experience in transit country | 1. Pleasant |
|  |  | 1. Unpleasant |
| Time in asylum center/process | Perceived experience in asylum center/asylum process | 1. Pleasant |
|  |  | 1. Unpleasant |
|  | Perceived emotions | 1. Positive emotion |
|  |  | 1. Negative emotion |
|  |  | 1. Mixed emotion |
|  | Barrier/difficulties | 1. Bureaucracy barrier |
|  |  | 1. Language barrier |
| Experiences in NL | Perceived experience after asylum | 1. Pleasant/positive |
|  |  | 1. Unpleasant/negative |
|  |  | 1. Mixed |
|  | Perceptions of Dutch people (non-government) | 1. Positive |
|  |  | 1. Negative |
|  | Perceptions of Dutch government/system | 1. Positive |
|  |  | 1. Negative |
|  | Opinion of NL | 1. Plus/Pros |
|  |  | 1. Mins/Cons |
|  | Experience of psychological and health problems | 1. Psychological problem |
|  |  | 1. Health/ physical problem |
| Coping strategy | Problem-focused | 1. Goal setting/planning |
|  |  | 1. Taking action (e.g. having structured daily activity, seeking help from family and friends, doing hobby or other beneficial activities) |
|  | Emotion-focused | 1. Cognitive reappraisal (positive) |
|  |  | 1. Acceptance/patience |
|  |  | 1. Others |
|  | Dysfunctional coping strategies | 1. Avoidance/distancing/denial |
|  |  | 1. Self-distraction (e.g. keep oneself busy to forget the problem) |
|  |  | 1. Self-blame |
| Factors contributing on resilience | Protective factors | 1. Social support |
|  |  | 1. Experience positive life events in past year |
|  |  | 1. Religiosity |
|  |  | 1. Maintaining cultural identity |
|  |  | 1. Meaning in life |
|  |  | 1. Others |
| Character of resilience people | Perceived character of resilience people | 1. Strong |
|  |  | 1. Patience/acceptance |
|  |  | 1. Having purpose/ life goal |
|  |  | 1. Self-determination/optimistic |
|  |  | 1. Creative |
|  |  | 1. Responsible |
|  |  | 1. Smart |
|  |  | 1. Others |
| Hope for the future | Intention to stay | 1. Stay, would not go back |
|  |  | 1. Stay, only back for a short visit |
|  |  | 1. Would go back someday, if safe |
|  |  | 1. Uncertain |
|  | Plans for future | 1. Continue studying (including learning language) |
|  |  | 1. Find a job/ start a business |
|  |  | 1. Buy own house |
|  |  | 1. Future for children |
|  |  | 1. Integrate to society |
|  |  | 1. Travelling |
|  |  | 1. Uncertain |
|  |  | 1. Get citizenship |
|  | Source of hope | 1. Family |
|  |  | 1. Self-efficacy |
|  |  | 1. Chances/ Opportunity from host country |
|  |  | 1. Others |
| Life Quote | Lesson learned from the difficulties | Lesson learned/ quote |
