# Supplementary material for: Adversity, emotion, and resilience among Syrian refugees in the Netherlands
Source: BMC Psychol. 2022 Nov 8;10:257. doi: 10.1186/s40359-022-00963-w (PMC9643972; doi:10.1186/s40359-022-00963-w)
Supplement: Supplementary file 3 — Additional file 3. The 32 Items COREQ Checklist. [file 40359_2022_963_MOESM3_ESM.docx]

**Consolidated criteria for reporting qualitative studies (COREQ): 32 items checklist**

| **No** | **Item** | **Guide questions/ description** | **Answers** |
| --- | --- | --- | --- |
| **Domain 1: Research team and reflexivity**  Personal Characteristics | | | |
| 1 | Interviewer | Which author/s conducted the interview? | The first author conducted the interviews. |
| 2 | Credentials | What were the researchers’ credentials? | The first author holds a master’s degree in Psychology, and the second and third authors hold Ph.D. |
| 3 | Occupation | What was their occupation at the time of the study? | The first author was a Ph.D. candidate, the second author was an associate professor, and the third author was a professor in social psychology. |
| 4 | Gender | Was the researcher male or female? | The first author, who conducted the interviews, was female. The other two researchers were female and male, respectively. |
| 5 | Experience and training | What experience or training did the researchers have? | The researchers have experience in working with refugees and minority groups, both in the research context and field. Moreover, the researchers are also trained in qualitative research methodology. |
| Relationship with participants | | |  |
| 6 | Relationship established | Was a relationship established prior to study commencement? | No, there was no relationship prior to the study. |
| 7 | Participant knowledge of the interviewer | What did the participants know about the researcher? (personal goals, reason for doing research) | The participants knew the reason and the purpose of the research, and also the background of the researcher. |
| 8 | Interviewer characteristics | What characteristics were reported about the interviewer? | The characteristic reported was a similar religious background to the majority of the participants. Moreover, the interviewer has experience working with refugees, which facilitated establishing a trusting relationship with the participants. |
| **Domain 2: Study design**  Theoretical framework | | |  |
| 9 | Methodological orientation and theory | What methodological orientation was stated to underpin the study? | Narrative research that relies on thematic analysis.  It is about how the parts of the participants’ stories are integrated to create a whole meaning. |
| Participant selection | | |  |
| 10 | Sampling | How were participants selected? | The participants were recruited using the snowball sampling technique based on personal networks and social media. |
| 11 | Method of approach | How were participants approached? | Two participants were personal contacts of a colleague, and the others responded to a research advertisement posted in a social media group for Syrian people in the Netherlands. The initial contact prior to the interview used email and personal chat room on social media. |
| 12 | Sample size | How many participants were in the study? | There were 18 participants in the study. |
| 13 | Non-participation | How many people refused to participate or dropped out? Reasons? | None. The participants signed up voluntarily and made it to the interviews. |
| Setting | | |  |
| 14 | The setting of data collection | Where was the data collected? | We used Skype for Business due to precautions relating to in-person meetings due to the COVID-19 pandemic. |
| 15 | Presence of non-participants | Was anyone else present besides the participants and researchers? | None. |
| 16 | Description of sample | What are the important characteristics of the sample? (demographic data) | The participants were Syrian refugees, ten men and eight women with a mean age of 29 years (age range 18-41, *SD*= 6.91). They had been in the Netherlands for an average of 3.2 years (*SD*= 2.2), with the average time spent in an asylum center being 7.2 months (*SD*= 5.51). Demographic information is shown in Table 1 in the manuscript. |
| Data collection | | |  |
| 17 | Interview guide | Were questions, prompts, and guides provided by the authors? Was it pilot tested? | Yes. |
| 18 | Repeat interviews | Were repeat interviews carried out? If yes, how many? | No. |
| 19 | Audio/ visual recording | Did the research use the audio or visual recording to collect the data? | We used the audio-visual recording to collect the data. Some participants opted for audio-only recording. |
| 20 | Field notes | Were field notes made during and/ or after the interview? | Yes. |
| 21 | Duration | What was the duration of the interviews? | The duration of the interviews ranged from 45 to 55 minutes. |
| 22 | Data saturation | Was data saturation discussed? | Yes. We stopped collecting the data after no new relevant knowledge was obtained from the participants. |
| 23 | Transcripts returned? | Were transcripts returned to participants for comments and/ or correction? | No. |
| **Domain 3: Analysis and findings**  Data analysis | | |  |
| 24 | Number of data coders | How many data coders coded the data? | Two independent coders: the first author and a trained research assistant. |
| 25 | Description of the coding tree | Did the authors provide a description of the coding tree? | Yes. |
| 26 | Derivation of themes | Were themes identified in advance or derived from the data? | The themes were identified from the data. The process of identifying the themes is described in the manuscript. |
| 27 | Software | What software, if applicable, was used to manage the data? | We used MAXQDA version 20 to manage the data. |
| 28 | Participant checking | Did participants provide feedback on the findings? | No. |
| Reporting | | |  |
| 29 | Quotations presented | Were participant quotations presented to illustrate the themes/ findings? Was each quotation identified? | Yes, and each quotation was identified. |
| 30 | Data and findings consistent | Was there consistency between the data presented and the findings? | Yes, there was consistency between the data presented and the findings. |
| 31 | Clarity of major themes | Were major themes clearly presented in the findings? | Yes. |
| 32 | Clarity of minor themes | Is there a description of diverse cases or a discussion of minor themes? | Yes. |
